# Revealing the beneficial effect of protease supplementation to high gravity beer fermentations using "-omics" techniques

**DOI:** 10.1186/1475-2859-10-27

**Published:** 2011-04-23

**Authors:** Maya P Piddocke, Alessandro Fazio, Wanwipa Vongsangnak, Man L Wong, Hans P Heldt-Hansen, Chris Workman, Jens Nielsen, Lisbeth Olsson

**Affiliations:** 1 Center for Microbial Biotechnology, Department of Systems Biology, Technical University of Denmark, DK-2800 Kongens Lyngby, Denmark; 2 Center for Biological Sequence Analysis, Department of Systems Biology, Technical University of Denmark, DK-2800 Kongens Lyngby, Denmark; 3 Chalmers University of Technology, Department of Chemical and Biological Engineering, SE-412 96 Gothenburg, Sweden; 4 Brewing and Alcoholic Beverage Department, Novozymes A/S, Denmark; 5 Center for Systems Biology, Soochow University, Suzhou 215006, China

## Abstract

**Background:**

Addition of sugar syrups to the basic wort is a popular technique to achieve higher gravity in beer fermentations, but it results in dilution of the free amino nitrogen (FAN) content in the medium. The multicomponent protease enzyme Flavourzyme has beneficial effect on the brewer's yeast fermentation performance during high gravity fermentations as it increases the initial FAN value and results in higher FAN uptake, higher specific growth rate, higher ethanol yield and improved flavour profile.

**Results:**

In the present study, transcriptome and metabolome analysis were used to elucidate the effect on the addition of the multicomponent protease enzyme Flavourzyme and its influence on the metabolism of the brewer's yeast strain Weihenstephan 34/70. The study underlines the importance of sufficient nitrogen availability during the course of beer fermentation. The applied metabolome and transcriptome analysis allowed mapping the effect of the wort sugar composition on the nitrogen uptake.

**Conclusion:**

Both the transcriptome and the metabolome analysis revealed that there is a significantly higher impact of protease addition for maltose syrup supplemented fermentations, while addition of glucose syrup to increase the gravity in the wort resulted in increased glucose repression that lead to inhibition of amino acid uptake and hereby inhibited the effect of the protease addition.

## Introduction

Despite the fact that beer brewing is a traditional process, there is in the today's competitive market a constant demand for further process improvement. One of the main targets for improvement in the brewing fermentation process is the fermentation time, wort fermentability and ethanol yield, equipment use and labor costs. High gravity brewing technique has been introduced since the 70s and gained increasing popularity in recent years as it meets many of these targets.

High gravity brewing uses wort with higher sugar concentration than the one normally used. The resulting beer has higher ethanol content, but the product is then diluted to the required alcohol content. A popular approach for achieving higher sugar concentration is the addition of sugar syrups as adjuncts to the wort, which usually has gravity in the range of 12-14°Plato. However, as sugar syrups do not contain any considerable amounts of nitrogen, their addition to the basic wort dilutes the free amino nitrogen (FAN) content present in the media. Thus, to ensure presence of sufficient nitrogen and to avoid incomplete brewer's yeast fermentation, it is a requirement that higher gravity wort has higher free amino nitrogen content compared to lower gravity wort. While FAN requirements for wort of 12°Plato are in the range of 140-150 mg l^-1^, wort with > 18°Plato requires FAN of 280 mg l^-1 ^[1]. To a certain extent, the FAN requirement is also strain specific [2].

In an earlier study we characterized high gravity beer fermentations at 21°Plato with increased available nitrogen content by addition of various nitrogen sources to the wort - ammonium sulphate, urea or proteases [3]. The results from this characterization showed that the nitrogen supplementation generated by the addition of the multicomponent enzyme (Flavourzyme) with both endo and exo peptidase activities resulted in the best fermentation performance in terms of higher ethanol yield, specific growth rate and specific ethanol productivity in addition to high FAN utilization.

In today's brewing market the efforts are focused on maintaining low sale prices despite an increase in raw material prices while maintaining profits. In order to reach these goals it is important to introduce innovative solutions, and the enzyme producing companies offer new solutions where enzymes can be used to improve the brewing process. The main targets for the currently available enzymes in brewing are focused on obtaining higher extract yields, improved attenuation control, longer beer filter cycle runs, shorter cooking cycles, and reduced beer losses and maturation time [4]. Proteases are commercially used for bioethanol production as a way to provide additional nitrogen available for the yeast. Protease addition to the simultaneous saccharification and fermentation of whole milled cereals is known to improve the final ethanol concentration and the volumetric productivity [5]. While addition of proteases is a commonly used practice for bioethanol production, studies of the addition of proteases as a way to enhance the free amino nitrogen in beer fermentation are very limited. In brewing, additions of proteases have further importance as the amino acid metabolism is related to the flavour and aroma profile of the final beer. The major classes of yeast derived flavour compounds are formed as by-products of the metabolism of sugars and amino acids [6]. Under fermentative conditions, some of the products from the amino acid metabolism might be released into the medium and contribute to the beer flavour.

To approach the problem with nitrogen limitations in high gravity beer fermentations, we showed that adding the multicomponent protease Flavourzyme gives superior fermentation performance in terms of increased initial FAN value and higher FAN uptake, higher specific growth rate, higher ethanol yield and improved flavour profile, in terms of lower acetaldehyde and ethyl acetate levels. In this study we therefore undertook a more detailed analysis of the effect of protease addition to high gravity worts using genome-wide transcriptome analysis and metabolome analysis.

## Materials and methods

### Strain

The flocculent bottom fermenting industrial lager beer yeast strain Weihenstephan 34/70 (Hefebank Weihenstephan, Freising, Germany) was used in this study. The strain was maintained as a frozen stock culture in 40% (v/v) glycerol.

### Fermentation setup

For the studied fermentations, wort with gravity corresponding to 21°Plato was used. Higher gravity was achieved with either glucose or maltose syrup supplementation to the basic wort of 14°Plato [7]. The fermentation setup was designed to simulate as closely as possible the larger scale beer fermentations.

### Wort

All-malt wort with starting gravity of 14.3°Plato and pH 5.2, (Alectia, Denmark), was used. This wort contained 90% carbohydrates of which the fermentable carbohydrates consisted of 4.4% fructose, 12.5% glucose, 66.5% maltose and 16.7% maltotriose (w/v). The wort also contained non-fermentable carbon sources such as dextrins and β-glucan. ZnSO_4_^.^7H_2_O, was added to a concentration of 0.1 ppm Zn. For adjusting the wort to higher gravity- 21°Plato, highly fermentable syrups- Clearsweet^® ^95% Refined Liquid Dextrose Corn Syrup (95.5% glucose, 2.5% maltose, 1% maltotriose, 1% higher saccharides, present in % dry basis (w/w) and Satin Sweet^® ^65% High Maltose Corn Syrup (70% maltose, 18% maltotriose, 9% higher saccharides, 2% glucose, present in % dry basis) were used as adjuncts [7]. Both syrups were kindly provided from Cargill Nordic A/S. Prior to inoculation, the wort was oxygenated with air until it reached 100% saturation.

### Fermentation conditions

For the pre-culture, the yeast from the stock culture was propagated on YPD plates at 30°C for four days. A single yeast colony was transferred to 20 ml of 14°Plato wort in a sterile 50 ml Falcon tube and incubated at 25°C in a rotary shaker at 150 rpm. After 48 hours, the preculture was transferred to a 500 ml shake flask with 375 ml of fresh wort and incubated for 72 h.

All fermentations were performed in 2.2 L bioreactor (Biostat B5; Braun Biotech International, Melsungen, Germany) with a working volume of 1.5 L. Dissolved oxygen was monitored with an autoclavable polarographic oxygen electrode. The fermentors were connected to Braun Biotech Multi-Fermenter Control System (MFCS) for data acquisition. Silicone based antifoam agent FD20P in a concentration of 0.1 ml/L (Basildon Chemicals, England) with food grade quality was used in the fermentations. The reactors were inoculated with a volume of pre-culture, corresponding to 1 × 10^7 ^cells/ml. During the cultivation the temperature was maintained at 14°C and the stirring was set to 90 rpm. Prior to sampling the stirring was increased to 300 rpm for 2 min. The higher stirring allowed better mixing and homogenization of the media and ensured representative sampling. The pH was recorded on-line, but not controlled. After the fermentation was completed, the whole fermentation broth was transferred to a sterile vessel and stored for 14 days at 0°C, for further maturation. Each of the studied fermentation conditions was investigated in duplicate experiments. Detailed physiological characteristics of the performed cultivations have been described previously [7].

### Calculations of growth characteristic parameters

The specific growth rate was determined as the slope from the linear function of the ln (natural logarithmic function) of the cell number (cells/ml) and the fermentation time (h) during the exponential growth phase. The yield coefficients were determined as the slope from the linear regression on the corresponding pairs of substrate (total saccharides) and product concentration (glycerol and ethanol).

### Protease supplementation

In order to increase the available free amino nitrogen content in the studied high gravity beer fermentations, the enzyme Flavourzyme was used as a supplement in a concentration of 60 ppm. Flavourzyme is a commercially available enzyme produced by Novozymes A/S. Previously, Flavourzyme has been commercially used to boost the nitrogen supplementation in bioethanol fermentations. It is a fungal multicomponent enzyme produced from *Aspergillus oryzae *with both endo- and exo- peptidase activities. The optimal temperature and pH for this enzyme complex is 50°C and pH 5-7, respectively.

### Enzyme hydrolysis

In order to determine the activity of the enzyme Flavourzyme and to investigate whether an increase in the gravity or change in the wort sugar composition influence its activity, the hydrolysis efficiency of Flavourzyme in wort at three different gravities was examined. The three different wort compositions were: 14 ˚Plato wort, 21 ˚Plato wort adjusted with glucose syrup and 21˚Plato wort adjusted with maltose syrup. The hydrolysis was performed in 1 L autoclaved shake flasks with a working volume of 800 ml of the selected wort. The enzyme Flavourzyme was added to each of the flask in a concentration of 60 ppm. As controls, shake flasks hydrolysis with no enzyme addition were used. The shake flasks were placed on a shaking table with an agitation of 90 rpm and a temperature of 14 ˚C, conditions that resemble those prevailing during the brewing experiment. For the sampling, 10 ml samples from each of shake flasks were taken daily. Each hydrolysis lasted for two weeks and was done in duplicates.

### Sampling

In order to ensure homogenous composition of the cultivation liquid, prior to each sampling, stirring was increased to 300 rpm. Both transcriptome and metabolome samples were stored at -80°C until further treatment. Samples for analysis of sugars and alcohols were collected on a regular basis every 24 hours throughout the fermentation. For measuring the free amino nitrogen content from the fermentations, samples were collected from the first and the final day of the primary fermentation. For all of the above analyses, 2-10 ml of fermentation samples were withdrawn from the fermentor, immediately filtered through a Cameo 0.20 μm pore size acetate/glass filters (Sartorius AG, Germany) and stored at -20°C prior to analysis.

For the cells count determination, samples were also collected daily. For the transcriptome analyses 20 ml samples were taken both from the early exponential and from the stationary phase. For the metabolome analyses, 10 ml samples were taken in the early phase and 20 ml samples, respectively were taken in the stationary phase. Each sampling was performed in duplicates.

### Free amino nitrogen (FAN)

The levels of free amino nitrogen (FAN) of the unfermented worts and from the last day of the beer fermentation were determined using the ninhydrin method at 570 nm [8]. Glycine was used as a standard.

### HPLC analysis

A Dionex Summit HPLC system (Synnyvale, CA) was used for analysis of sugars and metabolites from the extracellular medium. All metabolites were detected refractometrically (Waters 410 Differential Refractometer Detector, Millipore Corp., Milford, MA) after separation on an Aminex HPX-87H column (Biorad, Hercules, CA) at a temperature of 60°C using 5 mM H_2_SO_4 _as eluent. To allow the separation of the sugars with different degree of polymerization, two Aminex columns were mounted in serie with isocratic elution at 0.40 ml/min. External standards of maltotriose (DP3), maltose (DP2), glucose (DP1), fructose (DP1), glycerol and ethanol were used.

### Metabolome analysis

After withdrawal from the fermentor, the samples were rapidly quenched into 20 ml precooled (-40°C) 72% methanol. Cells were centrifuged at 10 000 × g for 20 minutes in -20°C to separate them from the quenching solution. Further on, the intracellular metabolites were extracted using chloroform: methanol: 3 mM Pipes buffer (pH = 7) extraction [9]. Following extraction, the samples were lyophilized using a Christ-Alpha 1-4 freeze dryer.

Following lyophilisation, the samples were resuspended in 200 μl of 1% (w/v) NaOH solution and derivatised using previously described methodology [10,11]. In order to decrease the matrix effect in the extracellular samples containing a high concentration of maltodextrins (sugars), the samples were resuspended in 2 ml of NaOH solution. As external standards, amino acid standards (Sigma) at two different levels were used. As internal standards, 20 mM of EDTA and 30 mM of chlorophenylalanine were used. Samples were normalised by the amount of intracellular standards and by the cell number and expressed as normalized peak area. Intra- and extracellular metabolites belonging to the group of amino acids and non-amino organic acids were analysed by GC-MS. GC-MS analysis was performed with a Hewlett-Packard system HP 6890 gas chromatograph coupled to a HP 5973 quadrupole mass selective detector (EI) operated at 70 eV. For the analyses, column J&W1701 column with size (30m ×250 μm × 0.15 μm) was used (Folsom, CA). The used GC-MS program has previously been described [12]. Peak detection was conducted with AMDIS (NIST, Gaithersburg, MD) using default parameters. Each sample was analysed in duplicates.

### Transcriptome analysis

#### Probe preparation and hybridization to arrays

Samples for RNA isolation were taken in duplicates during the stationary phase of the fermentations. For each sample, 20 mL of culture were sampled into 50 mL tubes containing 20 mL crushed ice and immediately centrifuged at 4000 rpm for 5 min at 4°C. The supernatant was discarded and the pellet was frozen instantaneously in liquid nitrogen and stored at -80°C. Total RNA was extracted using RNeasy Mini Kit (Qiagen), according to the protocol for total RNA isolation from yeast. The quality and the integrity of the extracted total RNA was analyzed using an Agilent Bioanalyzer 2100 (Agilent technologies Inc., USA) and RNA 6000 Nano LabChip kit. The cDNA and cRNA syntheses, labeling and cRNA hybridization on the oligonucleotide Yeast Genome 2.0 Array (Affymetrix, CA) were performed as described in the Affymetrix GeneChip^® ^expression analysis manual [13]. GeneChip^® ^Hybridization Oven, Fluidics Station FS-450 and 3000 7G Scanner were used for array hybridization, washing, staining and scanning.

#### Data acquisition and gene expression analysis

Affymetrix Microarray Suite v5.0 was used to generate CEL files from the scanned microarrays. Data analysis was performed by using the statistical open source language R [14]. Data preprocessing was carried out by using the Robust Multichip Average (RMA) method [15]. The function *rma *is contained in the R/affy package [16] and it implements RMA by correcting the Perfect Match (PM) probes, performing normalization [17] and calculating the expression measure by using median polish method.

In order to select genes whose expression levels were related to the experimental factors, Two-Way Analysis Of Variance (ANOVA) was performed. The 2×2 experimental design comprises two main factors ('wort type' and 'protease supplementation'), each having two levels ('glucose wort/maltose wort' and 'protease supplemented/protease non-supplemented'). The Two-Way ANOVA model was fitted in order to identify significantly changed gene expression levels with respect to the two above mentioned factors, as well as, the interaction term ('wort type*protease supplementation'). The P-value was corrected for multiple testing by applying the False Discovery Rate (FDR) methodology [18] and genes were selected by imposing a cut-off value of 0.05. Furthermore, as regards to the gene lists associated with the two main factors, only genes with |log_2_(fold change)| > 1.301 were considered. Genes selected within the interaction term were further investigated in order to find out which genes showed differences in each of the four possible factor level combinations: 'glucose wort*protease supplemented', 'glucose wort*protease non-supplemented', 'maltose wort*protease supplemented' and 'maltose wort*protease non-supplemented'. The identification of these genes was achieved by using the template match method and the R/code [19]. Moreover, gene ontology (GO) process terms of the selected genes were determined using GO Slim Mapper tools (Saccharomyces Genome Database (SGD)) with significance at P-value < 0.01. Normalized gene expression data was deposited at Gene Expression Omnibus (GEO) database [20] with accession number GPL2529 (platform), GSM607733-GSM607740 (samples) and GSE24645 (series).

#### Reporter Regulators (Transcription Factors) analysis

Reporter regulators, also named Reporter Transcription Factors or TFs, were determined using the software and regulatory network of Oliveira et al., 2008 [21]. The software is based on reconstructed graph covering each known transcription factor or regulatory protein, connected to all genes known to be effected by these proteins from the Yeast Protein Database (YPD).

In order to apply Reporter TFs, pairwise t-test analysis was performed to obtain statistical values. After that gene expression data sets was combined with reconstructed graph covering each known transcription factor or regulatory protein to identify a key transcription factor and its regulatory pathway that was the most significantly effected by the addition of protease in the 21°Plato glucose or maltose syrup supplemented fermentations.

## Results

During the course of high gravity beer fermentations, brewer's yeast is exposed to a number of stressful conditions such as high osmotic pressure caused by the high glucose concentrations in the beginning of the fermentations and ethanol stress, imposed by the elevated ethanol concentration levels towards the end of the fermentations. Furthermore, an increase in the gravity by the addition of sugar syrups to the media results in lower free amino nitrogen concentrations than the minimum required, thus brewer's yeast is exposed to an additional stress, caused by nitrogen limitation and limitations in the level of other nutrients, resulting in restricted growth [22]. In order to further investigate the effect of protease addition and its influence on the brewer's yeast metabolism, detailed intra and extracellular metabolome and transcriptome analyses were performed in samples collected from the early exponential and from the stationary phase of the fermentations.

### Enzyme hydrolysis

In order to determine the activity of the enzyme at the conditions prevailing during beer fermentation, enzyme hydrolysis experiments were performed.

**Figure 1:**
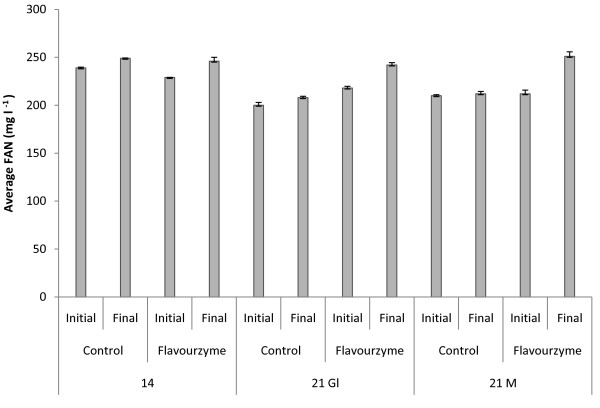
**Results from the FAN measurements of initial and final samples collected during enzyme hydrolysis assays**. The values are average of duplicate measurements with standard deviation < 10%.

With optimum temperature at 50°C and pH of 5-7, obviously, the lager beer fermentations at 14°C, with a pH ranging from around 5.2 to 4 during the course of the fermentations, provide harsh conditions for the enzyme. According to the Flavourzyme product sheet, at the conditions imposed during lager beer fermentation, the enzyme will be functioning with approximately 40% of its maximum activity. To determine the activity of Flavourzyme during the course of lager beer fermentations, Flavourzyme hydrolysis was performed at conditions resembling the conditions of lager beer fermentations. The results of the FAN analysis for the initial and the final sample of the enzyme hydrolysis experiments are presented in Figure 1.

The hydrolysis experiments showed significantly higher increase in the FAN content, for the Flavourzyme supplementation (25 to 40 mg l^-1^), while variations at the FAN value for the control fermentations remain within the measurement standard deviation of 10%.

### Physiological characterisation of the Flavourzyme supplemented fermentations

Previous characterisation of the Flavourzyme supplemented fermentations has shown to result in a higher specific growth rate, but a similar overall ethanol yield compared to the non supplemented fermentations [3].

**Figure 2:**
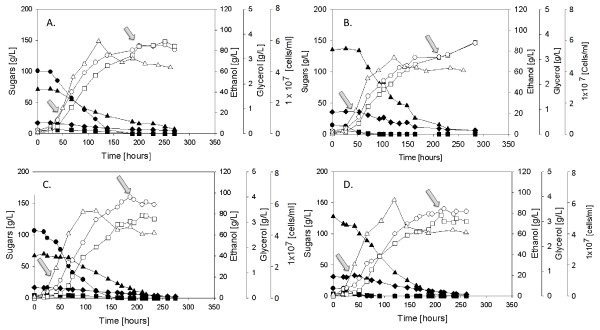
**A. Control fermentations with glucose syrup supplementation; B. Control fermentations with maltose syrup supplementation; C. Glucose syrup supplemented fermentations with Flavourzyme addition; D. Maltose syrup supplemented fermentations with Flavourzyme addition**. Arrows in the figures correspond to the sampling points for metabolome and transcriptome analysis of the studied fermentations. In the graphs, black circles represent the concentrations of glucose, black squares of fructose, black triangles of maltose, black diamonds of maltotriose, white squares of ethanol, white circles of glycerol and white triangles represent the numbers of cells/ml.

Both for the glucose and maltose supplemented fermentations with Flavourzyme addition, the specific growth rate was 0.074 (h^-1^), compared to 0.050 (h^-1^) and 0.064 (h^-1^) for the glucose and maltose syrup supplemented fermentations, respectively, without enzyme supplementations. For the glucose syrup supplemented fermentations, the ethanol yield was 0.48 g/g both for the non supplemented and Flavourzyme supplemented fermentations, while the glycerol yield was 0.026 and 0.024 (g/g), respectively. For the maltose supplemented fermentations, ethanol yield was 0.49 (g/g) for the control and 0.47 (g/g) for the Flavourzyme supplemented fermentations, while the glycerol yield remain the same in both cases (0.018 g/g). Furthermore, supplementation with Flavourzyme also resulted in shorter lag phase of the brewer's yeast cell growth. Despite the similar or slightly lower ethanol yield for the Flavourzyme supplemented fermentations, due to the higher specific growth rate, the specific ethanol productivity (calculated from the yield coefficients multiplied by the growth rate) of those fermentations was higher (Figure 2). The Flavourzyme supplemented fermentations also showed improved wort fermentability. While the non supplemented fermentations resulted in final gravity of 5.2 and 4.3, for the glucose and maltose syrup supplementation, respectively, the same fermentations with Flavourzyme addition had final gravity of 4.9 and 3.5, respectively, pointing to an improved utilization of the nitrogen available in the wort.

In order to further investigate the effect of the multicomponent enzyme Flavourzyme on the metabolism of the lager brewer's yeast strain Weihenstephan 34/70, following the physiological characterization, samples for transcriptome analysis and intra- and extracellular metabolome analysis from the early exponential phase (around 40 to 50 fermentation hours) and from the stationary phase (around 200 to 220 fermentation hours) were collected (the sampling points are indicated by arrows in Figure 2). The exact time of sampling for the studied fermentation conditions varied between the glucose and maltose syrup supplemented as well as between protease supplemented and non-supplemented fermentations.

### Metabolome analysis

Metabolites are the end products of cellular regulatory processes and they play a very important role in connecting many different pathways that operate within a living cell. Metabolome analysis is complicated since it includes all small molecules in a biological system. In addition, metabolites are heterogeneous, have different types of structures, functional groups, physicochemical properties, concentrations and many of them are still unknown [9]. Thus, the levels of metabolites can be regarded as the ultimate response of an organism to genetic alterations or environmental influences.

In the current study, the principle of alkylation reaction using methyl chloroformate [10,11] was used to enable simultaneous separation, detection and quantification of both intracellular and extracellular metabolites, belonging to the groups of amino acids and non-amino organic acids as well as their derivatives. From the approximately 600 metabolites included in the genome-wide metabolic reconstruction for yeast *Saccharomyces cerevisiae *[23], 40% are amines, amino acids and organic acids. Out of those, the applied method used in-house developed MS library, consisting of 75 metabolites which play a major role in the central carbon metabolism and amino acid biosynthesis. For calculating the fold change of the metabolites between the studied conditions, the value for each metabolite was represented as the average value from duplicate analysis of metabolome samples from the same fermentation as well as from duplicate fermentations.

#### Intracellular metabolites

In total, 39 intracellular metabolites were determined from the early exponential and from the stationary phase of the studied fermentations. In order to investigate the impact of the enzyme addition on the type of the sugar syrups used, the samples for the glucose and maltose syrups supplemented fermentations from the early exponential and from the stationary phase were compared.

Comparison between the glucose and maltose syrup supplemented fermentations with protease supplementation showed higher concentration for most of the amino acids and organic acids for the maltose syrup supplemented fermentations with protease addition (See Table 1 and Table 2). The effect on the addition of Flavourzyme was especially pronounced for the early exponential phase of the studied fermentations. From the early exponential phase of the control fermentations only asparagine was present in higher level for maltose syrup supplemented fermentations. For the samples from the early exponential phase, Flavourzyme addition in the maltose syrup supplemented fermentation showed an increase in the fold change for fifteen of the studied amino acids (Table 1). The fold change increase for most of the amino acids was in the range from 1 to 2, with the exception of isoleucine, that had a fold change increase of 2.74. For the stationary phase of the control fermentations, in total, also fifteen amino acids were present in higher fold change for the maltose syrup supplemented fermentations, with a fold change in the range from 1 to 13.8. For the stationary phase of the control fermentations, higher fold change for the maltose syrup supplemented fermentations compared to the glucose syrup supplemented ones, was observed for the intermediate of cysteine- cystathionine (13.8), histidine (13.2), phenylalanine (10.7) and lysine (8.95) (Table 1).

**Table 1:** Fold change of the intracellular amino acids and amino acid intermediates compounds of the maltose syrup versus glucose syrup supplemented fermentations

	Maltose/Glucose Early exponential phase		Maltose/Glucose Stationary phase	
	
	Control	Flavourzyme supplementation	Control	Flavourzyme supplementation
**Alanine**	0.19	1.18	4.63	1.22

**Glycine**	0.05	0.22	5.46	1.40

**2-aminobutyric acid**	0.13	0.49	0.29	1.61

**Valine**	0.12	1.15	6.06	1.49

**Leucine**	0.14	1.97	3.61	1.35

**Isoleucine**	0.38	2.74	4.01	0.95

**4-amino-n-butyric acid**	-	1.20	1.41	0.85

**Proline**	0.13	0.82	0.55	1.11

**Threonine**	0.42	1.00	0.89	0.39

**Serine**	0.26	1.75	1.44	0.73

**Asparagine**	1.02	1.37	1.82	1.00

**Methionine**	0.74	1.78	-	-

**N-acetyl-L-glutamate**	-	-	0.37	0.54

**Phenylalanine**	0.29	1.40	10.7	1.14

**Trans-4-hydroxyproline**	-	-	-	1.87

**Ornithine**	-	0.35	1.02	0.90

**Lysine**	0.35	0.65	8.95	1.99

**Glutamine**	-	-	-	2.50

**Histidine**	0.29	1.31	13.2	1.03

**Tyrosine**	0.50	1.01	3.50	0.84

**Tryptophan**	0.42	1.14	7.38	1.19

**Cystathionine**	0.27	0.46	13.8	1.29

**Aspartic acid**	0.27	1.10	3.26	1.69

**Glutamic acid**	0.19	1.76	3.72	1.00

The underlined numbers represents an increase in the fold change between the Flavourzyme supplemented versus non-supplemented fermentations for the respective fermentation phase. The symbol "-"indicates that the metabolites were not present in the studied conditions.

**Table 2:** Fold change of the intracellular organic acids for the studied fermentations of the maltose syrup versus glucose syrup supplemented fermentations

	Maltose/Glucose Early exponential phase		Maltose/Glucose Stationary phase	
	**Control**	**Flavourzyme supplementation**	**Control**	**Flavourzyme supplementation**
	
**Pyruvic acid**	-	3.63	-	0.68

**Malonic acid**	-	0.13	-	0.35

**(3S)-3-Methyl-2-oxopentanoic acid**	-	0.36	-	0.61

**Fumaric acid**	-	1.16	-	0.96

**Malic acid**	-	1.16	-	0.96

**Succinic acid**	0.10	1.57	1.36	0.62

**Citramalic acid**	0.00	1.03	1.69	0.45

**Nicotinic acid**	0.29	3.09	0.34	1.10

**2-isopropylmalic acid**	-	-	0.39	1.30

**2-oxoglutaric acid**	-	0.84	0.49	2.70

**Citric acid**	0.20	1.74	1.46	1.59

**cis-Aconitic Acid**	0.35	1.98	3.75	1.27

**Pyroglutamic acid**	0.26	-	-	-

**Isocitric acid**	0.09	0.64	0.10	1.23

**Cumaric acid**	-	0.71	-	0.56

**5-hydroxymethyl-2-furaldehyde**	-	0.78	-	0.46

**Glutaric acid**	-	0.01	-	0.71

**Glyceric acid**	-	1.71	-	0.91

The underlined numbers represent the increase in the fold change for the studied metabolites between the Flavourzyme supplemented versus non-supplemented fermentations for the respective fermentation phase. The symbol "-"indicates that the metabolites were not present in the studied conditions.

All of the studied organic acids from the early exponential phase of the control fermentations were present in higher concentrations for the glucose syrup supplemented fermentations compared to the maltose syrup supplemented ones. Addition of Flavourzyme to the maltose syrup supplemented fermentations resulted in increase in the concentrations for most of the TCA cycle intermediates. While for most of the organic acids the increase in the fold change was in the range between 1 to 2, pyruvic acid and nicotinic acid showed higher fold change increase to 3.63 and 3.09, respectively. In the stationary phase, the control fermentations with maltose syrup supplementation resulted in higher fold increase in the range of 1.36 to 3.72 for succinic, citramalic, citric and cis-aconitic acids, respectively. Addition of Flavourzyme to the maltose supplemented fermentations resulted in higher fold change for nicotinic, isocitric, cis-aconitic, 2-isopropylmalic, citric and 2-oxoglutaric acids. Their fold change increase was in the range of 1.10 to 2.70.

#### Extracellular metabolites

In total, 14 extracellular metabolites were determined from the early exponential and from the stationary phase of the studied fermentations. In general, fewer compounds were found extracellularly than intracellularly.

Comparison of the determined extracellular metabolites from the stationary phase between the maltose and glucose syrup supplemented fermentations without protease supplementation, showed significantly higher concentrations of the amino acids and organic acids involved in the pyruvate metabolism- alanine, valine, leucine, pyruvic acid and 2-isopropylmalic acid. In addition, phenylalanine and tyrosine involved in the phosphoenolpyruvate metabolism and isoleucine were also present in significantly higher concentrations for the maltose syrup supplemented fermentations (Table 3). For the glucose supplemented fermentations without protease supplementation, the TCA cycle intermediates- citric acid, succinic acid, cis-aconitic acid and 2-oxoglutaric acids were present in significantly higher concentrations.

**Table 3:** Fold change of the extracellular amino and organic acids detected from the stationary phase of the studied fermentations

	Maltose/Glucose		Glucose/Maltose	
	**Control**	**Flavourzyme supplementation**	**Control**	**Flavourzyme supplementation Alanine**
	
**Alanine**	> 20	1.97		0.51

**Valine**	> 20	2.09	-	0.48

**Leucine**	> 20	9.36	-	0.11

**Isoleucine**	> 20	9.36	-	0.11

**Proline**	2.51	0.29	0.40	3.43

**Citric acid**	0.95	0.52	1.05	1.94

**Phenylalanine**	> 20	1.48	-	0.67

**Tyrosine**	> 20	> 20	-	-

**Sucinic acid**	0.45	0.23	2.24	4.28

**Cis-Aconitic Acid**	-	-	> 20	6.62

**2-oxoglutaric acid**	-	-	> 20	-

**Pyruvic acid**	> 20	0.36	-	2.79

**Citramalic acid**	-	-	-	> 20

**2-isopropylmalic acid**	> 20	-	-	-

The underlined numbers represent the increase in the fold change for the respective metabolites between the Flavourzyme supplemented versus non-supplemented fermentations. The symbol "-"indicates that the compound was not present in at least one of the studied conditions.

Addition of Flavourzyme to the maltose syrup supplemented fermentations resulted in further increase of the alanine, valine, leucine, isoleucine and phenylalanine concentrations (Table 3). Addition of Flavourzyme to the glucose syrup supplemented fermentations resulted in further increase of the concentrations of the TCA intermediates- citric, succinic, pyruvic acid as well as increase in the concentration of citramalic acid.

### Transcriptome analysis

To study the effect on the addition of the multicomponent protease Flavourzymes on the metabolism of the brewer's yeast Weihenstephan 34/70, genome-wide transcription profiles from the stationary phase of the studied fermentations were analyzed. To quantitatively determine the genes with significantly changed expression, Two-Way ANOVA analysis was performed.

Based only on the type of sugar syrup used (maltose rich versus glucose rich), at a P-value of 0.05, Two-Way ANOVA test revealed 311 genes with significant changes in their expression. Of those, 204 were up-regulated and 107 were down-regulated in the maltose syrup supplemented fermentations. The levels of the fold change for the significantly changed genes were in the range from -4 to 3.4. Among the up-regulated genes, the most significantly enriched GO terms revealed overrepresentation for the following categories: organelle organization, RNA metabolic process, translation, transcription, transport, cell cycle as well as response to stress, carbohydrate and lipid metabolism (Additional file 1). Of the up-regulated genes, 6 genes, (representing 2.9% of cluster frequency) were involved in the amino acids related metabolism and protein catabolism. The six genes were involved in amino acids biosynthesis (*ECM17*), glycine catabolism (*GRS1*), branch chain amino acids- leucine, isoleucine, valine synthesis and catabolism (*ILV6*, *ARO80*), tyrosine (*MSY1*) and phenylalanine (*MSF1*) synthesis. For the down-regulated genes, the GO overrepresented terms included transport, response to stress, RNA metabolic process, organelle organization and transcription as well as amino acid and amino acid derivatives metabolic processes, protein catabolic processes, carbohydrate and lipid metabolism. In total, 9 genes, representing 8.4% cluster frequencies, were involved in the amino acid and protein catabolic processes (Additional file 2: Table S1). Those genes were mainly involved in the glutamate (*GDH3*, *CIT2*), histidine (*HIS1*), methionine (*ADI1*, *STR3*) and asparagine (*ASN1*) biosynthesis as well as glycine (*GCV1*) and proline (*PUT1*) catabolism.

To schematically represent the parts of the brewer's yeast metabolism where sugar syrup addition and protease supplementation resulted in most significant transcriptional changes based on the Two Way ANOVA analysis, the genome scale metabolic model of *S. cerevisiae iIN800 *was used [24]. Overview of the yeast metabolic network including the significantly changed genes based on the effect of sugar syrup addition (glucose versus maltose) is presented in Additional file 1.

Based on the effect of the enzyme addition itself, at a P-value of 0.05, Two-Way ANOVA test of the four studied conditions revealed 169 significantly changed genes. Of those genes, 87 were down-regulated and 82 were up-regulated in the presence of Flavourzyme.

The GO overrepresented terms for the down-regulated genes included translation, organelle organization, RNA metabolic processes, ribosome biogenesis, transport, transcription and cell cycle (Additional file 3: Table S2). Six of those genes were involved directly in amino acid metabolism. They encode the biosynthesis of leucine (*LEU2*), histidine (*HIS1*, *HTS1*), lysine (*LYS12*), isoleucine (*ILV3*) and phenylalanine-t-RNA synthetase (*MSF1*), respectively.

For the up-regulated genes, the GO terms included transport, organelle organization, response to stress, RNA metabolic process, protein modification process, transcription, DNA metabolic process.

Among them, the genes involved in the amino acid metabolism were cystathionine beta-lyase (*STR3*), converting cystathionine into homocysteine, proline oxidase (*PUT1*) and acireductone dioxygenease (*ADI1*), involved in the methionine salvage pathway (Additional file 3: Table S2). Overview of the yeast metabolic network including the significantly changed genes based on the effect of enzyme addition is presented in Additional file 4.

**Figure 3:**
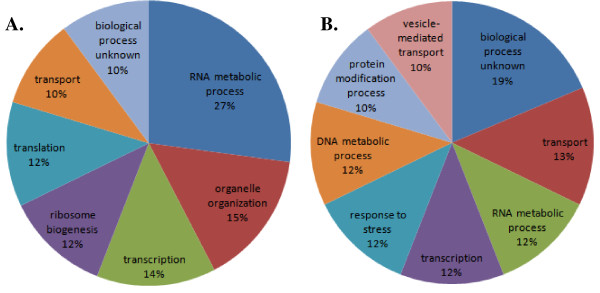
**Schematic overview of the GO annotation, based on the biological process ontology, for the significantly changed genes in maltose syrup supplemented fermentations with Flavourzyme addition**. **A**. The main overrepresented GO annotation categories for the up regulated genes. **B**. The main overrepresented GO annotation categories for the down regulated genes.

At a p-value of 0.05, the interaction effect, accounting for both the addition of Flavourzyme and the type of sugar syrup used to increase the gravity, resulted in significant change of the transcriptional response for only 6 genes for the glucose supplemented fermentations, while for the maltose supplemented fermentations, in total, 86 genes were significantly changed. Two of the significantly changed genes for the glucose syrup supplemented fermentations were putative proteins with unknown function, *YJR011C *and *YCR016W*, respectively. The four genes with known function- *RME1*, *MCD4*, *SLD2 *and *SWD2 *were involved in cell growth related processes such as transcription, cell cycle, meiosis, RNA and DNA metabolic processes (Figure 3). Among the four genes, only *MCD4 *was up-regulated while the rest of the genes were down-regulated.

Among the 86 significantly changed genes for the maltose supplemented fermentations with Flavourzyme addition, 41 genes were down-regulated and 45 genes were up-regulated. In order to determine significantly enriched Gene Ontology (GO) process terms within the significantly changed genes, based on the interaction effect of the type of sugar syrup and addition of protease, Saccharomyces Genome Database (SGD) - GO tools with significance at p < 0.05 were used. The most overrepresented GO terms among the up-regulated genes with known function were transport and cell growth related processes such as RNA metabolic process, transcription and DNA metabolic process (Additional file 5: Table S3). 11 of the 45 up-regulated genes were ORF with unknown function. Three of the up-regulated genes - *ARG82*, *ARO80 *and *MET13*, were directly involved in amino acid metabolism. *ARG82 *is inositol polyphosphate multikinase (IPMK), regulating arginine-, phosphate-, and nitrogen-responsive genes; *ARO80 *is activating transcription of aromatic amino acid catabolic genes in the presence of aromatic amino acids and *MET13 *is major isozyme of methylenetetrahydrofolate reductase, involved in the methionine biosynthesis pathway. Among the down-regulated genes, the most overrepresented GO terms were also cell growth related processes such as RNA metabolic process, organelle organization, transcription, ribosome biogenesis and translation. Three of the down-regulated genes- *FRS2*, *VAS1*, *HTS1 *were also involved in cellular amino acid and derivative metabolic process. *FRS2 *is alpha subunit of cytoplasmic phenylalanyl-tRNA synthetase, *VAS1 *is mitochondrial and cytoplasmic valyl-tRNA synthetase and *HTS1 *is cytoplasmic and mitochondrial histidine tRNA synthetase.

**Figure 4:**
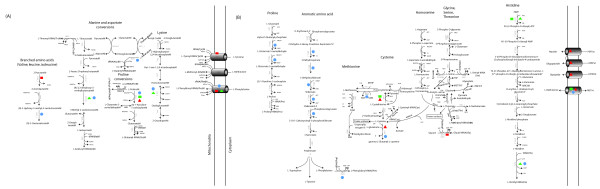
**Parts of *Weihenstephan 34/70 *amino acid metabolism (based on the genome scale metabolic model of *S. cerevisiae iIN800*) with significantly changed genes in the metabolic network identified from Two Way ANOVA Analysis**. The significantly expressed genes based on the effect of type of sugar added (maltose versus glucose) are represented by square. The significantly expressed genes based on the effect of enzyme added (enzyme versus non-enzyme supplementation) are represented by triangle. Shapes coloured in red represent the up-regulated genes and shapes coloured in green represent the down-regulated genes for the respective studied effect. Blue circle represents significantly expressed genes of the interaction effect (sugar*enzyme).

To schematically represent the parts of the brewer's yeast amino acid metabolism where the interaction effect of protease supplementation and type of sugar syrup used as a supplement resulted in most significant transcriptional changes based on the Two Way ANOVA analysis (Figure 4), the genome scale metabolic model of *S. cerevisiae iIN800 *was used [24]. Detailed overview of the most significant transcriptional changes in the brewer's yeast amino acid metabolism as a result of the interaction effect of protease supplementation and sugar syrup used as adjunct is presented in Additional file 6.

Detailed yeast metabolic map including all significantly changed genes from Two Way ANOVA analysis for the interaction effect (sugar*enzyme supplementation) in the brewer's yeast metabolic network (based on the genome scale metabolic model *S.cerevisiae iIN800*) is presented in Additional file 7.

#### Analysis of Regulators

To identify the transcription factors around which the most significant changes occur, the reporter features algorithm [21], using the interaction based on protein-protein interaction networks was applied (Table 4, hypergeometric test at P < 0.01). In total, the analysis revealed 5 TFs with high degree of transcriptional regulation.

**Table 4:** Transcription factors for the maltose supplemented fermentations, based on the t-test comparison between the control and Flavourzyme supplemented fermentations

Transcription factors	Number of neighbours	Z-score	P-value
**FKS1**	24	2,7	0,003
**POS5**	141	2,48	0,007
**HAP1**	24	2,47	0,008
**MOT3**	19	2,34	0,01
**UPC2**	17	2,08	0,02

The number of neighbours indicates the number of genes regulated by the particular transcription factor. Z-score represents the score of each TF, calculated based on the score of its neighbours. The P-value gives measure of significance and all results ≤0.05 are reported.

Among those, Fks1 is involved in cell wall synthesis and maintenance, Pos5 is mitochondrial NADH kinase and phosphorylates NADH and NAD(+) with lower specificity and it is required for the response to oxidative stress, Hap1 is a zinc finger transcription factor involved in the complex regulation of gene expression in response to levels of heme and oxygen, Mot3 is nuclear transcription factor involved in repression of a subset of hypoxic genes by Rox1p, repression of several *DAN*/*TIR *genes during aerobic growth, and repression of ergosterol biosynthetic genes. While Hap1 and Mot3 are involved in RNA metabolic processes and transcription, Pos5 is involved in response to stress and cofactor metabolic process, Hap1 in cellular respiration, Fks1 in cell wall and membrane organization and transport and Upc2 in carbohydrate metabolic process. To account for the directionality of the reported regulations, Reporter TFs for only the up-regulated or only the down-regulated genes were performed. Among the five significantly altered TFs, only Mot3 was involved in the regulation of the down-regulated genes, while the rest of the TFs were involved in the regulation of the up-regulated genes.

## Discussion

In the food industry, enzymes can be used as alternatives for traditional chemical-based technology and can substitute the use of synthetic chemicals in many different process applications. Their advantages are associated with more specific modes of action, reduced formation of byproducts and as a result, improved environmental performance of the production process such as lower energy consumption and biodegradability of waste products.

Here we show that addition of the multicomponent enzyme Flavourzyme led to increase in the available FAN in wort, which in turn resulted in improved fermentation performance with higher specific growth rate and more favored flavour profile of the final beer. In addition to the importance of the assimilable nitrogen availability, our detailed metabolome and transcriptome analysis confirmed the importance of the wort sugar composition on the utilization of the supplied nitrogen. Both transcriptome and metabolome analysis revealed significantly lower impact on the Flavourzyme addition for the glucose syrup supplemented fermentations compared to the maltose ones.

Thus, the difference in the amino acids and organic acid profile among the studied fermentations were driven by the combined effect of two major events. On one side, the intracellular metabolome profile revealed higher intracellular concentration for most of the amino acids in the maltose syrup supplemented fermentations compared to the glucose ones. Increase in the range of 1-2 fold for the amino acids in the early exponential phase for the maltose syrup supplemented fermentations revealed that addition of Flavourzyme primarily influenced the amount of nitrogenous compounds in the maltose syrup supplemented wort. During the stationary phase, comparison across the metabolome profile between the glucose and maltose syrup supplemented fermentations without addition of proteases also showed higher concentration for most of the amino acids and some of the organic acids for the stationary phase samples of the maltose syrup supplemented fermentations. Furthermore, this difference was further enhanced with the addition of Flavourzyme.

The amino acids present in wort have been previously classified into three groups based on the impact formation of α-keto acid analogues that represent important flavour compounds [25]. According to this classification, aspartate, asparagine, glutamate, glutamine, threonine, serine, methionine and proline belong to class 1, isoleucine, valine, phenylalanine, glycine, alanine and tyrosine belong to class 2 and lysine, histidine, arginine and leucine belong to class 3. While the concentration of amino acids from class 1 in the wort is considered to be relatively unimportant, as they can be either assimilated from the wort or synthesized *de novo*, the initial concentration of the compounds from class 2 is crucial since in the later stages of the fermentation the synthesis of these compounds from sugars is repressed. The amino acids from class 3 are also of importance since they are delivered exclusively from the wort. Thus, deficiencies in the amino acids from group 2 and group 3 will restrict the synthesis of compounds derived from the α-keto acid analogue of these amino acids and metabolism of their related by-products and thus affect the beer quality [6]. Addition of Flavourzyme leads to an increase in the amino acid content mainly present in class 2 and 1, as well as histidine belonging to class 3. Thus, addition of Flavourzyme might also contribute to a more balanced flavour profile of the resulting beer product.

Addition of Flavourzyme to the maltose syrup supplemented fermentations revealed intracellular increase in the amino acids such as alanine, valine and leucine involved in the pyruvate and phosphoenol pyruvate metabolism both from the early exponential and from the stationary phase. Comparison across the glucose and maltose syrup fermentations without and with Flavourzyme addition also revealed higher concentrations of these amino acids for the maltose syrup supplemented fermentations. Increase in the pyruvic acid itself, as well as most of the other TCA cycle intermediates such as fumaric, malic, succinic and citric acid from the early exponential phase of the maltose syrup supplemented fermentations was also observed. The increase in the concentrations of those compounds is possibly an indicator of the higher metabolic activity in the maltose syrup supplemented fermentations compared to the glucose ones [5]. Addition of Flavourzyme to the glucose syrup supplemented fermentations results in an increase in the intracellular concentrations of TCA cycle intermediates such as 2-oxoglutaric acid, citric acid and isocitric acid in the stationary phase. The observed accumulation of TCA intermediates inside the cell could be a result of amino acids catabolism, but it may also be due to repression on yeast growth due to the increased osmotic pressure leading to incomplete TCA cycle metabolism [6].

Transcriptome analyses confirmed the higher impact of Flavourzyme supplementation on the maltose syrup supplemented fermentations compared to the glucose ones. While statistical analyses revealed only 6 significantly changed genes upon addition of Flavourzyme for the glucose syrup supplemented fermentations, 86 genes were significantly changed for the maltose syrup supplemented fermentations.

Addition of Flavourzyme did not result in the same transcriptional response in the glucose syrup supplemented fermentations compared to the maltose syrup supplemented ones. Presence of high concentrations of glucose in the growth medium likely represses the transcription of multiple genes involved in the alternative carbohydrate and mitochondrial metabolism. This phenomenon is known as carbon catabolite repression (CCR). CCR encounter coordinated down regulation of the transcription of large group of genes involved in metabolism of non-glucose carbon sources, a number of hexose transporters and respiration [26,27]. A major role in the global regulation of CCR is played by the two nutrient signaling transducers- Snf1 and Gcn2. Tor1 is another important nutritional transducer which has been implicated in the up-regulation of the general amino acid permease with broad specificity- Gap1, in the down regulation of the tryptophan and tyrosine permease- Tat2 and the high affinity histidine permease- Hip1. A study of Peter et al., 2006 [28] found that transport of neutral, cationic and anionic amino acids is regulated by CCR at the protein expression and functional levels. By deletion of various genes involved in the amino acids sensing and uptake, the authors proved that signaling of the activation of neutral and cationic amino acid permeases due to CCR activation is via the TOR1 pathway and not through the Snf1/Mig1, Gcn2 or Ras kinase pathways. The authors observed an increase in the amino acid transporter activity for all three classes of amino acids when *S. cerevisiae *was grown on alternative carbon source as for example galactose media, compared to those grown on glucose media. This observation may explain the lower amino acid uptake for the glucose syrup supplemented fermentations with Flavourzyme addition, where only a small increase in the relative concentration for some of the studied intracellular amino acids (in our metabolome profile) and low number of significantly changed genes (in our transcriptome profile) were observed compared to maltose syrup supplemented fermentations with Flavourzyme addition. Thus, it is possible that the observed carbon catabolite repression in glucose rich media also represses the uptake of nitrogenous compounds in the wort. As a result of such repression, the low number of significantly changed genes in the glucose syrup supplemented fermentations is possible indication that the regulatory control of the Flavourzyme addition is mainly achieved at the levels of enzyme kinetics (metabolome) levels and not at hierarchical (transcriptome) levels [29].

On the contrary, for the maltose syrup supplemented fermentations, addition of Flavourzyme might have also resulted in significant changes at the levels of transcription/translation and post-translational modifications.

## Conclusion

In conclusion, changes in the amino acid uptake when glucose or alternative to glucose carbon sources are used, plays an important role in protein synthesis and other processes of cell metabolism. For example, amino acids form intermediates for the major catabolic pathways such as acetyl CoA, pyruvate and 2-oxoglutarate [28]. Therefore, the wort's amino acid content and utilization in combination with the wort's sugar composition used is of great importance since it will have significant impact, not only on the brewer's yeast metabolism, but also on the flavour profile of the final beer. This study is also an illustration how combination of transcriptome and metabolome analyses can be used for "system-wide" analysis in industrial yeast fermentations for better understanding of the complex secondary metabolism and the types of regulation (hierarchical and/or metabolic) as a result of given environmental change.

## Declaration of competing interests

The authors declare that they have no competing interests.

## Authors' contributions

MPP- carried out most of the experimental work, data interpretation and drafted the manuscript; AF - performed analysis of the transcriptome data; WV- performed analysis of the transcriptome data and draw the metabolic maps; MLW - carried out part of the fermentation studies, transcriptome and metabolome sampling; HPHH- participated in the discussion of the data and manuscript correction; CW- manuscript correction; JN- discussion of the data and manuscript correction; LO- discussion and interprepation of the data, manuscript correction and edited the manuscript. All authors read and approved the final manuscript.

## Supplementary Material

Additional file 1 **Overview of the brewer's yeast metabolic network including the significantly changed genes based on the effect of sugar syrup addition**. The figure presents parts of the brewer's yeast metabolism where sugar syrup supplementation resulted in most significant transcriptional changes based on the Two Way ANOVA analysis. Click here for file

Additional file 2 **GO annotation based on the biological process ontology for the significantly changed genes in glucose versus maltose syrup supplemented fermentations regardless of the Flavourzyme addition**. Table S1 contains the most overrepresented categories, from the GO annotation based on the biological process ontology, including the significantly changed up- and down-regulated genes for the glucose versus maltose syrup supplemented fermentations regardless of the Flavourzyme addition. Click here for file

Additional file 3 **GO annotation based on the biological process ontology for the significantly changed genes from the comparison of enzyme (Flavourzyme) supplemented versus non-enzyme (control) fermentations, regardless of the type of sugar syrup used as adjunct**. Table S2 includes the most overrepresented categories from the GO annotation (based on the biological process ontology) for the significantly changed up- and down-regulated genes for the Flavourzyme supplemented versus non supplemented fermentations, regardless of the type of sugar syrup used as adjunct. Click here for file

Additional file 4 **Overview of the brewer's yeast metabolic network including the significantly changed genes based on the effect of enzyme addition**. The figure presents a schematic overview of the brewer's yeast metabolism where protease supplementation resulted in most significant transcriptional changes based on Two Way ANOVA analysis. Click here for file

Additional file 5 **GO annotation based on the biological process ontology for the significantly changed genes in maltose syrup supplemented fermentations with Flavourzyme addition**. Table S3 presents the overrepresented GO annotation categories, based on the biological process ontology, for the significantly changed up- and down-regulated genes in maltose syrup supplemented fermentations with Flavourzyme addition. Click here for file

Additional file 6 **Overview of the brewer's yeast amino acid metabolism (based on the genome scale metabolic model of *S. cerevisiae iIN800*) including the significantly changed genes of the interaction effect on enzyme and sugar syrup addition**. The figure presents a close-up look of the brewer's yeast amino acid metabolism where the interaction effect of enzyme and sugar syrup addition results in most significant transcriptional changes. Click here for file

Additional file 7 **Overview of the brewer's yeast metabolic network including the significantly changed genes from the interaction effect of protease supplementation and sugar syrup addition**. The figure presents a schematic overview of the brewer's yeast metabolism where the interaction effect of enzyme and sugar syrup addition results in most significant transcriptional changes. Click here for file
