# Supplementary material for: Revealing the beneficial effect of protease supplementation to high gravity beer fermentations using "-omics" techniques
Source: Microb Cell Fact. 2011 Apr 23;10:27. doi: 10.1186/1475-2859-10-27 (PMC3107165; doi:10.1186/1475-2859-10-27)
Supplement: Additional file 2 — GO annotation based on the biological process ontology for the significantly changed genes in glucose versus maltose syrup supplemented fermentations regardless of the Flavourzyme addition. Table S1 contains the most overrepresented categories, from the GO annotation based on the biological process ontology, including the significantly changed up- and down-regulated genes for the glucose versus maltose syrup supplemented fermentations regardless of the Flavourzyme addition. [file 1475-2859-10-27-S2.DOC]

**Table S1. GO annotation based on the biological process ontology for the significantly changed genes in glucose versus maltose syrup supplemented fermentations regardless of the Flavourzyme addition.**

| **GO term** | **Gene hits** | **Cluster Frequency** | **GO term** | | **Gene hits** | **Cluster Frequency** |
| --- | --- | --- | --- | --- | --- | --- |
| ***Up-regulated genes (107)*** |  |  | ***Down- regulated genes (204)*** | |  |  |
| **transport** | 20 | 18.7% | | **organelle organization** | 52 | 25.5% |
| **response to stress** | 18 | 16.8% | | **RNA metabolic process** | 45 | 22.1% |
| **RNA metabolic process** | 16 | 15% | | **translation** | 39 | 19.1% |
| **biological process unknown** | 16 | 15% | | **biological process unknown** | 34 | 16.7% |
| **organelle organization** | 15 | 14.0% | | **transport** | 27 | 13.2% |
| **transcription** | 14 | 13.1% | | **transcription** | 20 | 9.8% |
| **response to chemical stimulus** | 14 | 13.1% | | **protein modification process** | 20 | 9.8% |
| **protein modification process** | 10 | 9.3% | | **cell cycle** | 17 | 8.3% |
| **cellular amino acid and derivative metabolic process** | 9 | 8.4% | | **ribosome biogenesis** | 16 | 7.8% |
| **Table S1,** *continued***.** GO annotation based on the biological process ontology for the significantly changed genes in glucose versus maltose syrup supplemented fermentations regardless of the Flavourzyme addition. | | | | | | |
| **carbohydrate metabolic process** | 8 | 7.5% | | **response to stress** | 15 | 7.4% |
| **generation of precursor metabolites and energy** | 7 | 6.5% | | **response to chemical stimulus** | 14 | 6.9% |
| **protein catabolic process** | 7 | 6.5% | | **DNA metabolic process** | 14 | 6.9% |
| **lipid metabolic process** | 7 | 6.5% | | **cofactor metabolic process** | 11 | 5.4% |
|  |  |  | | **carbohydrate metabolic process** | 9 | 4.4% |
|  |  |  | | **membrane organization** | 9 | 4.4% |
|  |  |  | | **lipid metabolic process** | 8 | 3.9% |

*Gene hits represent the number of genes for the up- or down regulated genes belonging to the particular GO term. This value is also given as percentage (cluster frequency).*
