# Supplementary material for: Revealing the beneficial effect of protease supplementation to high gravity beer fermentations using "-omics" techniques
Source: Microb Cell Fact. 2011 Apr 23;10:27. doi: 10.1186/1475-2859-10-27 (PMC3107165; doi:10.1186/1475-2859-10-27)
Supplement: Additional file 3 — GO annotation based on the biological process ontology for the significantly changed genes from the comparison of enzyme (Flavourzyme) supplemented versus non-enzyme (control) fermentations, regardless of the type of sugar syrup used as adjunct. Table S2 includes the most overrepresented categories from the GO annotation (based on the biological process ontology) for the significantly changed up- and down-regulated genes for the Flavourzyme supplemented versus non supplemented fermentations, regardless of the type of sugar syrup used as adjunct. [file 1475-2859-10-27-S3.DOC]

**Table S2.** GO annotation based on the biological process ontology for the significantly changed genes based on the comparison of enzyme (Flavourzyme) versus non-enzyme (control) supplementation, regardless of the type of sugar syrup used as adjunct.

| **GO term** | **Gene hits** | **Cluster Frequency** | **GO term** | **Gene hits** | **Cluster Frequency** |
| --- | --- | --- | --- | --- | --- |
| ***Up-regulated genes (82)*** |  |  | ***Down- regulated genes (87)*** |  |  |
| **biological process unknown** | 19 | 23.2% | **translation** | 26 | 29.9% |
| **transport** | 16 | 19.5% | **organelle organization** | 25 | 28.7% |
| **organelle organization** | 13 | 15.9% | **RNA metabolic process** | 23 | 26.4% |
| **response to stress** | 11 | 13.4% | **ribosome biogenesis** | 14 | 16.1% |
| **RNA metabolic process** | 10 | 12.2% | **transport** | 13 | 14.9% |
| **protein modification process** | 9 | 11% | **transcription** | 9 | 10.3% |
| **transcription** | 7 | 8.5% | **cell cycle** | 7 | 8.0% |
| **DNA metabolic process** | 7 | 8.5% | **biological process unknown** | 7 | 8.0% |
| **response to chemical stimulus** | 6 | 7.3% | **cellular amino acid and derivative metabolic process** | 6 | 6.9% |
| **cofactor metabolic process** | 6 | 7.3% | **cofactor metabolic process** | 5 | 5.7% |
| **vesicle-mediated transport** | 6 | 7.3% | **carbohydrate metabolic process** | 4 | 4.6% |
| **Table S2** *continued*. GO annotation based on the biological process ontology for the significantly changed genes based on the comparison of enzyme (Flavourzyme) versus non-enzyme (control) supplementation, regardless of the type of sugar syrup used as adjunct. | | | | | |
| **generation of precursor metabolites and energy** | 5 | 6.1% | **response to chemical stimulus** | 4 | 4.6% |
| **lipid metabolic process** | 5 | 6.1% | **protein modification process** | 4 | 4.6% |
| **signal transduction** | 5 | 6.1% | **response to stress** | 4 | 4.6% |

*Gene hits represent the number of genes for the up- or down regulated genes belonging to the particular GO term. This value is also given as percentage (cluster frequency).*
