# Supplementary material for: Revealing the beneficial effect of protease supplementation to high gravity beer fermentations using "-omics" techniques
Source: Microb Cell Fact. 2011 Apr 23;10:27. doi: 10.1186/1475-2859-10-27 (PMC3107165; doi:10.1186/1475-2859-10-27)
Supplement: Additional file 5 — GO annotation based on the biological process ontology for the significantly changed genes in maltose syrup supplemented fermentations with Flavourzyme addition. Table S3 presents the overrepresented GO annotation categories, based on the biological process ontology, for the significantly changed up- and down-regulated genes in maltose syrup supplemented fermentations with Flavourzyme addition. [file 1475-2859-10-27-S5.DOC]

**Table S3.** GO annotation based on the biological process ontology for the significantly changed genes in maltose syrup supplemented fermentations with Flavourzyme addition.

| **GOID** | **GO term** | **Gene hits** | **Cluster Frequency** |
| --- | --- | --- | --- |
| ***Up-regulated genes (45)*** |  |  |  |
|  | biological process unknown | 11 | 24.4% |
|  | transport | 8 | 17.8% |
|  | RNA metabolic process | 7 | 15.6% |
|  | transcription | 7 | 15.6% |
|  | response to stress | 7 | 15.6% |
|  | DNA metabolic process | 7 | 15.6% |
|  | protein modification process | 6 | 13.3% |
|  | vesicle-mediated transport | 6 | 13.3% |
| ***Down- regulated genes (41)*** |  |  |  |
|  | RNA metabolic process | 16 | 39.0% |
|  | organelle organization | 9 | 22% |
|  | transcription | 8 | 19.5% |
|  | ribosome biogenesis | 7 | 17.1% |
|  | translation | 7 | 17.1% |
|  | transport | 6 | 14.6% |
|  | biological process unknown | 6 | 14.6% |

*Gene hits represent the number of genes for the up- or down regulated genes belonging to the particular GO term. This value is also given as percentage (cluster frequency).*
