# Supplementary figures and images for: Revealing the beneficial effect of protease supplementation to high gravity beer fermentations using "-omics" techniques
Source: Microb Cell Fact. 2011 Apr 23;10:27. doi: 10.1186/1475-2859-10-27 (PMC3107165; doi:10.1186/1475-2859-10-27)

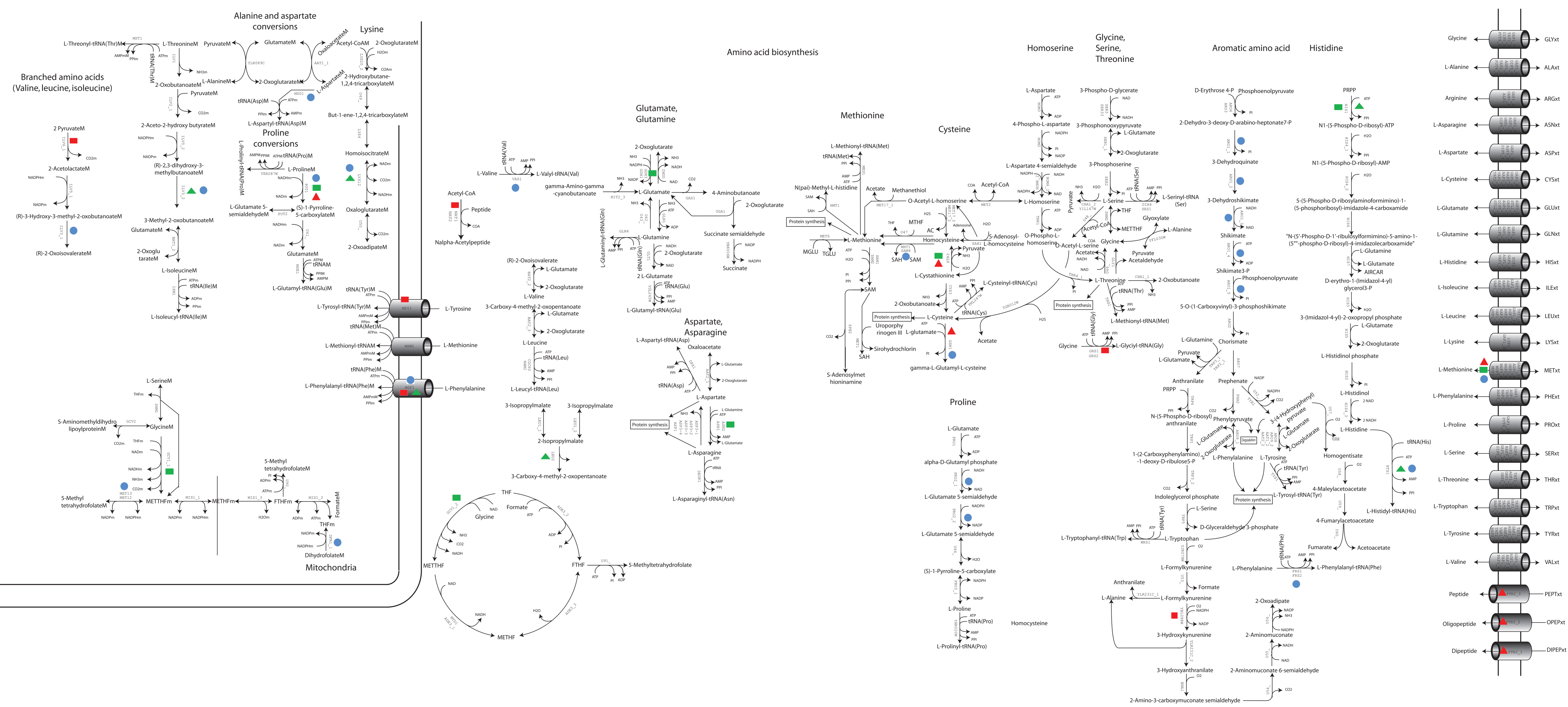

Supplement: Additional file 6 — Overview of the brewer's yeast amino acid metabolism (based on the genome scale metabolic model of S. cerevisiae iIN800) including the significantly changed genes of the interaction effect on enzyme and sugar syrup addition. The figure presents a close-up look of the brewer's yeast amino acid metabolism where the interaction effect of enzyme and sugar syrup addition results in most significant transcriptional changes. [file 1475-2859-10-27-S6.PDF]
