# Supplementary material for: Revealing the beneficial effect of protease supplementation to high gravity beer fermentations using "-omics" techniques
Source: Microb Cell Fact. 2011 Apr 23;10:27. doi: 10.1186/1475-2859-10-27 (PMC3107165; doi:10.1186/1475-2859-10-27)

# S. cerevisiae genome-scale metabolic model iIN800

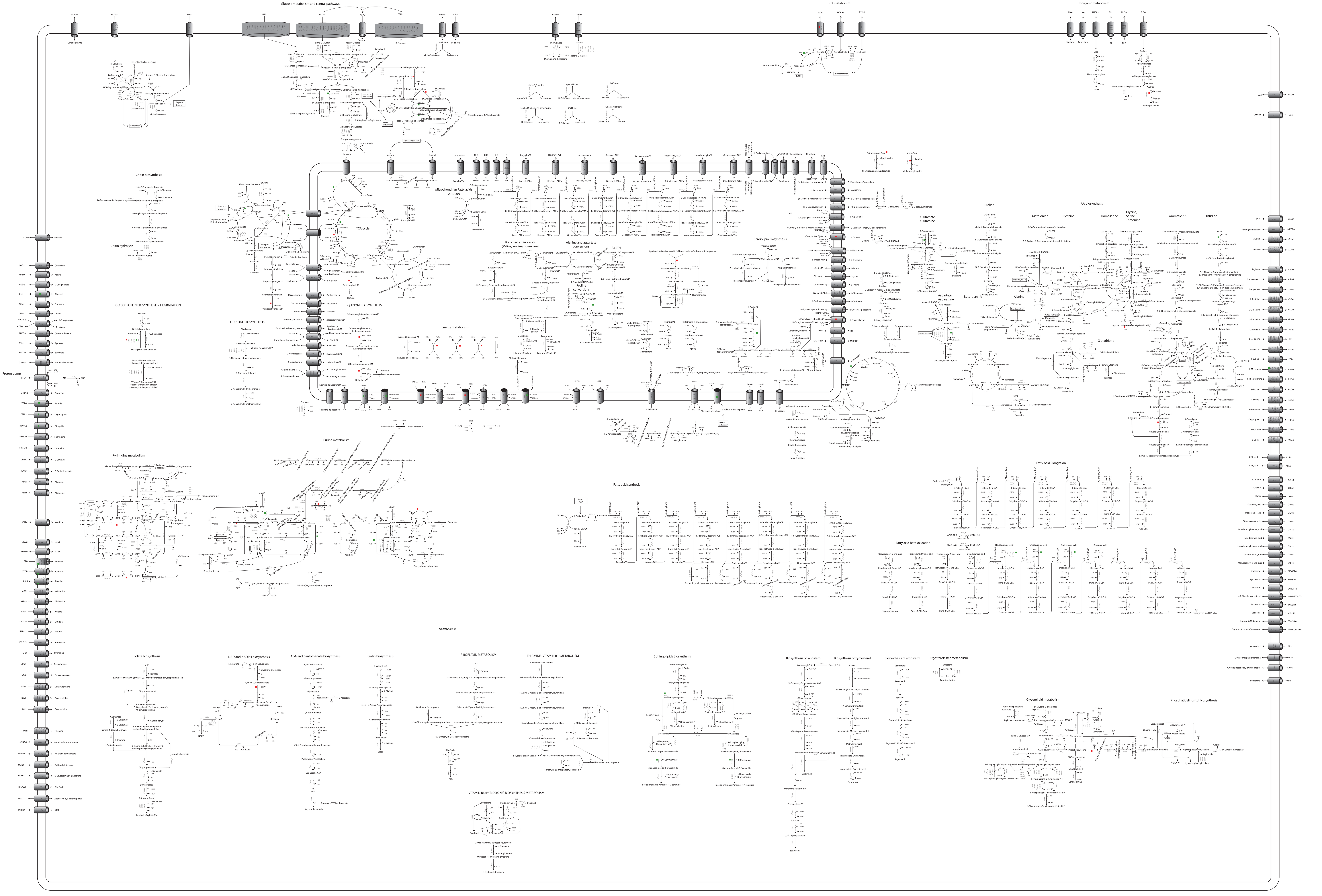

Supplement: Additional file 1 — Overview of the brewer's yeast metabolic network including the significantly changed genes based on the effect of sugar syrup addition. The figure presents parts of the brewer's yeast metabolism where sugar syrup supplementation resulted in most significant transcriptional changes based on the Two Way ANOVA analysis. [file 1475-2859-10-27-S1.PDF]
