# Supplementary material for: Revealing the beneficial effect of protease supplementation to high gravity beer fermentations using "-omics" techniques
Source: Microb Cell Fact. 2011 Apr 23;10:27. doi: 10.1186/1475-2859-10-27 (PMC3107165; doi:10.1186/1475-2859-10-27)

S. cerevisiae genome-scale metabolic model iIN800

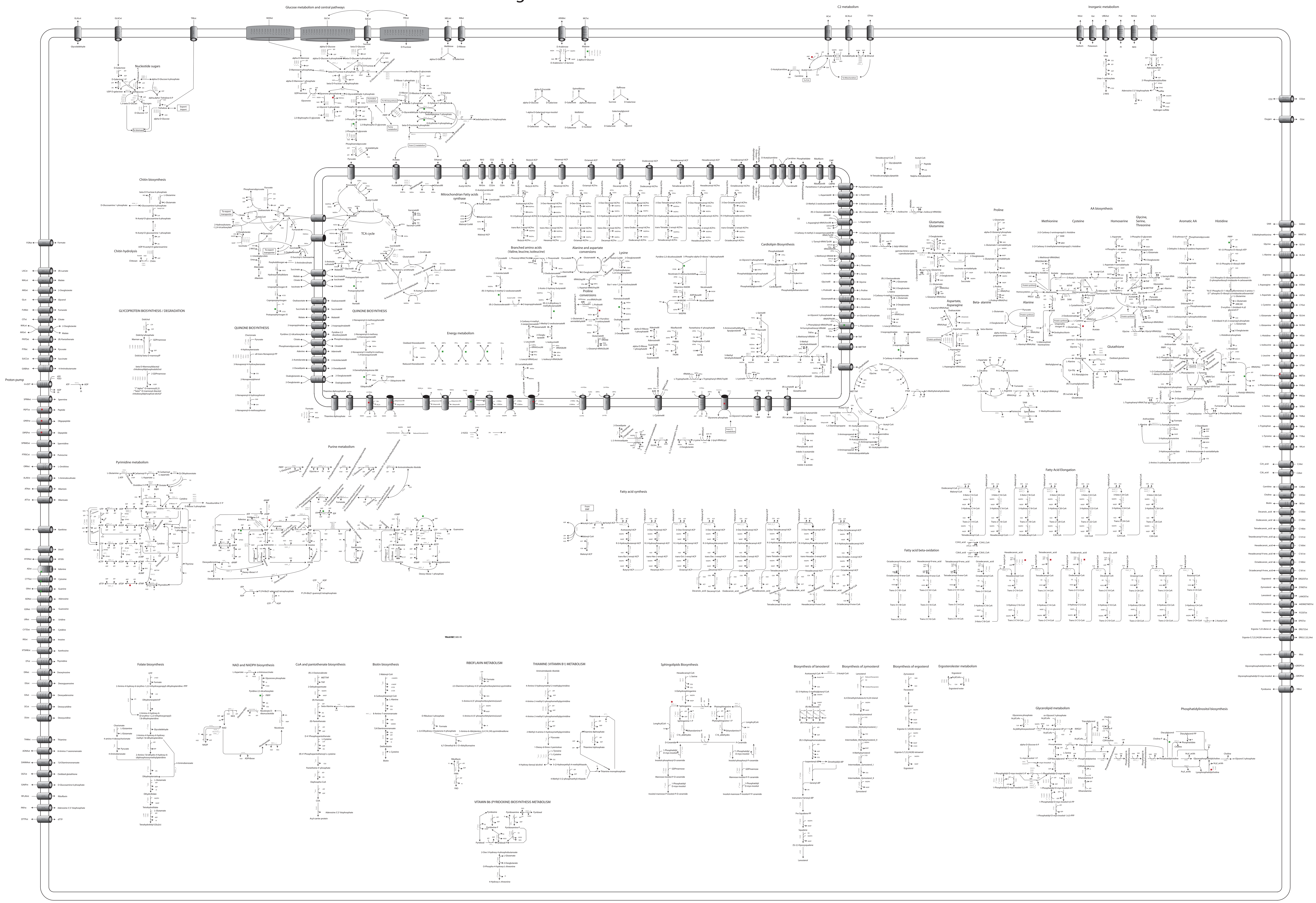

Supplement: Additional file 4 — Overview of the brewer's yeast metabolic network including the significantly changed genes based on the effect of enzyme addition. The figure presents a schematic overview of the brewer's yeast metabolism where protease supplementation resulted in most significant transcriptional changes based on Two Way ANOVA analysis. [file 1475-2859-10-27-S4.PDF]
