# Supplementary material for: Revealing the beneficial effect of protease supplementation to high gravity beer fermentations using "-omics" techniques
Source: Microb Cell Fact. 2011 Apr 23;10:27. doi: 10.1186/1475-2859-10-27 (PMC3107165; doi:10.1186/1475-2859-10-27)

S. cerevisiae genome-scale metabolic model iIN800

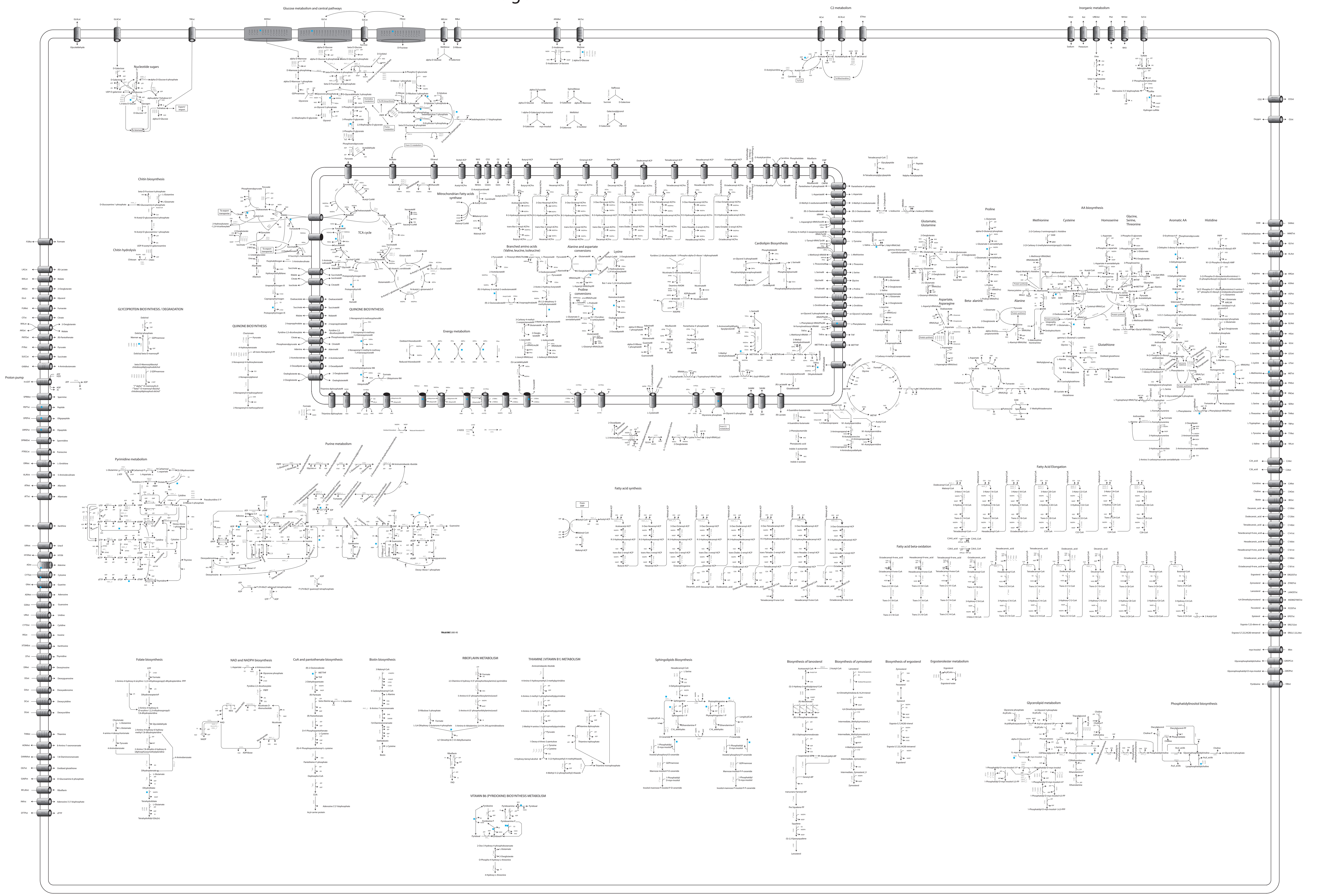

Supplement: Additional file 7 — Overview of the brewer's yeast metabolic network including the significantly changed genes from the interaction effect of protease supplementation and sugar syrup addition. The figure presents a schematic overview of the brewer's yeast metabolism where the interaction effect of enzyme and sugar syrup addition results in most significant transcriptional changes. [file 1475-2859-10-27-S7.PDF]
